# Supplementary material for: Hot spot prediction in protein-protein interactions by an ensemble system
Source: BMC Syst Biol. 2018 Dec 31;12(Suppl 9):132. doi: 10.1186/s12918-018-0665-8 (PMC6311905; doi:10.1186/s12918-018-0665-8)
Supplement: Supplementary file 2 — All relevant MATLAB code. A simple Matlab implement of our predictor. (ZIP 19,742 kb) [file 12918_2018_665_MOESM2_ESM.zip › Additional file 2/Read ASA/ASA website/dbMPIKT/4BJ5C.html]

 NetSurfP - Protein Surface Accessibility and Secondary Structure Predictions

  

|  |  |  |
| --- | --- | --- |
|  |  | NetSurfP - Protein Surface Accessibility and Secondary Structure Predictions  Technical University of Denmark |

  


---

```
# For publication of results, please cite:
# A generic method for assignment of reliability scores applied to solvent accessibility predictions.
# Bent Petersen, Thomas Nordahl Petersen, Pernille Andersen, Morten Nielsen and Claus Lundegaard
# BMC Structural Biology 2009, 9:51 doi:10.1186/1472-6807-9-51
#
# Column 1: Class assignment - B for buried or E for Exposed - Threshold: 25% exposure, but not based on RSA
# Column 2: Amino acid
# Column 3: Sequence name
# Column 4: Amino acid number
# Column 5: Relative Surface Accessibility - RSA
# Column 6: Absolute Surface Accessibility
# Column 7: Z-fit score for RSA prediction
# Column 8: Probability for Alpha-Helix
# Column 9: Probability for Beta-strand
# Column 10: Probability for Coil
E G  4BJ5C                  1    0.875  68.831  -1.375   0.003   0.003   0.994
E A  4BJ5C                  2    0.612  67.420  -1.999   0.018   0.019   0.964
E S  4BJ5C                  3    0.543  63.593  -1.518   0.053   0.043   0.903
E N  4BJ5C                  4    0.531  77.709  -1.213   0.053   0.043   0.903
E S  4BJ5C                  5    0.616  72.172  -0.284   0.053   0.043   0.903
B Y  4BJ5C                  6    0.239  51.181   0.468   0.052   0.084   0.864
E A  4BJ5C                  7    0.461  50.769   0.021   0.052   0.084   0.864
B I  4BJ5C                  8    0.104  19.259   0.641   0.053   0.043   0.903
B P  4BJ5C                  9    0.273  38.710  -0.051   0.053   0.043   0.903
E E  4BJ5C                 10    0.592 103.510  -0.229   0.115   0.016   0.868
E N  4BJ5C                 11    0.468  68.471  -0.881   0.181   0.016   0.803
E E  4BJ5C                 12    0.481  84.066  -0.183   0.268   0.043   0.689
B L  4BJ5C                 13    0.162  29.571  -0.324   0.268   0.043   0.689
B L  4BJ5C                 14    0.151  27.557   0.683   0.268   0.043   0.689
E D  4BJ5C                 15    0.424  61.041   0.183   0.430   0.016   0.555
E E  4BJ5C                 16    0.543  94.845   0.063   0.782   0.003   0.216
E D  4BJ5C                 17    0.460  66.344  -0.435   0.858   0.002   0.139
B T  4BJ5C                 18    0.087  12.136  -0.243   0.858   0.002   0.139
B M  4BJ5C                 19    0.180  35.998   0.161   0.858   0.002   0.139
E N  4BJ5C                 20    0.495  72.527   0.771   0.858   0.002   0.139
B F  4BJ5C                 21    0.089  17.963   0.341   0.923   0.002   0.076
B I  4BJ5C                 22    0.048   8.972  -0.734   0.923   0.002   0.076
E S  4BJ5C                 23    0.350  41.067  -0.657   0.923   0.002   0.076
B S  4BJ5C                 24    0.274  32.148  -0.826   0.858   0.002   0.139
B L  4BJ5C                 25    0.047   8.624   0.151   0.858   0.002   0.139
E K  4BJ5C                 26    0.321  66.071   0.432   0.858   0.002   0.139
E N  4BJ5C                 27    0.493  72.175  -0.563   0.782   0.003   0.216
B D  4BJ5C                 28    0.198  28.517  -0.912   0.782   0.003   0.216
B L  4BJ5C                 29    0.063  11.554  -0.859   0.782   0.003   0.216
E S  4BJ5C                 30    0.415  48.603   0.192   0.694   0.003   0.303
E N  4BJ5C                 31    0.572  83.799   0.744   0.694   0.003   0.303
B I  4BJ5C                 32    0.152  28.157  -0.330   0.502   0.002   0.495
E S  4BJ5C                 33    0.436  51.052  -0.835   0.321   0.003   0.675
E N  4BJ5C                 34    0.611  89.494  -1.591   0.181   0.016   0.803
E S  4BJ5C                 35    0.629  73.719  -0.981   0.058   0.017   0.925
B L  4BJ5C                 36    0.175  32.042   0.358   0.018   0.019   0.964
E P  4BJ5C                 37    0.437  62.024  -0.395   0.018   0.019   0.964
B F  4BJ5C                 38    0.257  51.560  -0.422   0.053   0.043   0.903
E E  4BJ5C                 39    0.601 104.977  -0.062   0.184   0.043   0.773
B Y  4BJ5C                 40    0.135  28.849   0.716   0.181   0.016   0.803
B P  4BJ5C                 41    0.222  31.544   0.103   0.522   0.016   0.462
E H  4BJ5C                 42    0.582 105.920   0.241   0.858   0.002   0.139
E E  4BJ5C                 43    0.442  77.182   0.910   0.923   0.002   0.076
B I  4BJ5C                 44    0.042   7.751   0.356   0.970   0.001   0.030
B A  4BJ5C                 45    0.096  10.612   0.086   0.970   0.001   0.030
E E  4BJ5C                 46    0.494  86.372   1.008   0.970   0.001   0.030
B A  4BJ5C                 47    0.217  23.935   0.363   0.970   0.001   0.030
B I  4BJ5C                 48    0.039   7.307   0.159   0.923   0.002   0.076
E R  4BJ5C                 49    0.331  75.730   0.842   0.782   0.003   0.216
E S  4BJ5C                 50    0.522  61.143  -0.829   0.502   0.002   0.495
E D  4BJ5C                 51    0.403  58.116  -0.047   0.339   0.016   0.645
B F  4BJ5C                 52    0.081  16.317  -0.096   0.257   0.016   0.727
E S  4BJ5C                 53    0.493  57.791   0.085   0.113   0.043   0.844
E N  4BJ5C                 54    0.400  58.516  -0.353   0.268   0.043   0.689
E E  4BJ5C                 55    0.466  81.340  -0.042   0.354   0.048   0.598
E D  4BJ5C                 56    0.570  82.079  -0.434   0.386   0.097   0.517
E I  4BJ5C                 57    0.395  73.001   0.488   0.386   0.097   0.517
B Y  4BJ5C                 58    0.178  37.953  -0.005   0.278   0.093   0.628
E D  4BJ5C                 59    0.497  71.647  -0.612   0.191   0.086   0.723
E N  4BJ5C                 60    0.550  80.535  -1.478   0.113   0.043   0.844
B I  4BJ5C                 61    0.161  29.859   0.489   0.052   0.084   0.864
E D  4BJ5C                 62    0.427  61.588   0.326   0.018   0.019   0.964
B P  4BJ5C                 63    0.262  37.121  -0.794   0.018   0.019   0.964
E D  4BJ5C                 64    0.738 106.375  -1.575   0.018   0.047   0.935
E T  4BJ5C                 65    0.468  64.884  -0.343   0.018   0.088   0.893
B I  4BJ5C                 66    0.099  18.296   0.238   0.019   0.141   0.840
E S  4BJ5C                 67    0.511  59.831  -0.706   0.019   0.141   0.840
B F  4BJ5C                 68    0.139  27.817  -0.036   0.018   0.047   0.935
B P  4BJ5C                 69    0.248  35.220  -0.086   0.018   0.019   0.964
B P  4BJ5C                 70    0.234  33.261  -0.794   0.058   0.017   0.925
E K  4BJ5C                 71    0.519 106.820   0.027   0.053   0.043   0.903
B I  4BJ5C                 72    0.123  22.774  -0.756   0.113   0.087   0.800
B A  4BJ5C                 73    0.195  21.489  -0.516   0.191   0.086   0.723
E T  4BJ5C                 74    0.326  45.244  -0.675   0.278   0.093   0.628
B T  4BJ5C                 75    0.134  18.614   0.043   0.455   0.046   0.498
E D  4BJ5C                 76    0.326  47.034  -0.758   0.561   0.047   0.393
B L  4BJ5C                 77    0.153  28.106  -0.241   0.522   0.016   0.462
B F  4BJ5C                 78    0.055  11.059   0.257   0.717   0.014   0.269
B L  4BJ5C                 79    0.055  10.089   0.923   0.802   0.014   0.185
B P  4BJ5C                 80    0.202  28.635  -0.082   0.802   0.014   0.185
B L  4BJ5C                 81    0.180  33.013  -0.059   0.802   0.014   0.185
B F  4BJ5C                 82    0.060  11.982  -0.167   0.802   0.014   0.185
B F  4BJ5C                 83    0.112  22.539  -0.068   0.717   0.014   0.269
E H  4BJ5C                 84    0.332  60.427  -0.066   0.522   0.016   0.462
B F  4BJ5C                 85    0.108  21.736  -0.496   0.339   0.016   0.645
E G  4BJ5C                 86    0.317  24.964  -0.869   0.339   0.016   0.645
B S  4BJ5C                 87    0.271  31.785  -0.745   0.339   0.016   0.645
B T  4BJ5C                 88    0.126  17.532  -0.592   0.802   0.014   0.185
E R  4BJ5C                 89    0.391  89.608   0.492   0.923   0.002   0.076
E Q  4BJ5C                 90    0.530  94.587   1.278   0.923   0.002   0.076
B F  4BJ5C                 91    0.043   8.710   0.092   0.970   0.001   0.030
B M  4BJ5C                 92    0.059  11.806   0.109   0.970   0.001   0.030
E D  4BJ5C                 93    0.526  75.840   1.113   0.970   0.001   0.030
E K  4BJ5C                 94    0.306  62.944   1.668   0.988   0.000   0.012
B L  4BJ5C                 95    0.025   4.614   0.650   0.970   0.001   0.030
E H  4BJ5C                 96    0.314  57.044   0.609   0.970   0.001   0.030
E E  4BJ5C                 97    0.518  90.512   1.249   0.970   0.001   0.030
B V  4BJ5C                 98    0.082  12.557   0.256   0.938   0.007   0.055
B I  4BJ5C                 99    0.058  10.785   0.200   0.879   0.010   0.111
E S  4BJ5C                100    0.535  62.749   0.462   0.522   0.016   0.462
E G  4BJ5C                101    0.464  36.533   0.225   0.181   0.016   0.803
E D  4BJ5C                102    0.570  82.123  -0.544   0.113   0.043   0.844
B Y  4BJ5C                103    0.159  33.914   0.349   0.053   0.043   0.903
E E  4BJ5C                104    0.517  90.285   0.405   0.058   0.017   0.925
E P  4BJ5C                105    0.481  68.183  -0.365   0.339   0.016   0.645
E S  4BJ5C                106    0.481  56.420  -0.887   0.502   0.002   0.495
E Q  4BJ5C                107    0.382  68.225   0.156   0.782   0.003   0.216
B A  4BJ5C                108    0.091   9.984  -0.986   0.858   0.002   0.139
E E  4BJ5C                109    0.426  74.422   0.220   0.879   0.010   0.111
E K  4BJ5C                110    0.404  83.185   1.153   0.938   0.007   0.055
B L  4BJ5C                111    0.063  11.517   0.813   0.938   0.007   0.055
B V  4BJ5C                112    0.065   9.960   0.293   0.879   0.010   0.111
E Q  4BJ5C                113    0.438  78.173   1.020   0.879   0.010   0.111
E D  4BJ5C                114    0.308  44.368  -0.253   0.802   0.014   0.185
B L  4BJ5C                115    0.041   7.599   0.422   0.717   0.014   0.269
B C  4BJ5C                116    0.239  33.570   0.214   0.502   0.002   0.495
E D  4BJ5C                117    0.516  74.413  -0.548   0.622   0.015   0.363
E E  4BJ5C                118    0.430  75.208  -0.272   0.622   0.015   0.363
B T  4BJ5C                119    0.108  15.021  -0.448   0.522   0.016   0.462
B G  4BJ5C                120    0.245  19.313  -0.981   0.430   0.016   0.555
B I  4BJ5C                121    0.097  17.963   0.035   0.455   0.046   0.498
E R  4BJ5C                122    0.290  66.433   0.596   0.455   0.046   0.498
E K  4BJ5C                123    0.400  82.198   0.299   0.561   0.047   0.393
E N  4BJ5C                124    0.330  48.341  -0.304   0.561   0.047   0.393
B F  4BJ5C                125    0.079  15.755   0.246   0.660   0.049   0.291
B S  4BJ5C                126    0.120  14.017  -1.029   0.660   0.049   0.291
B T  4BJ5C                127    0.216  30.001  -0.629   0.751   0.050   0.199
B S  4BJ5C                128    0.148  17.346  -0.355   0.831   0.044   0.125
B I  4BJ5C                129    0.041   7.585   0.140   0.831   0.044   0.125
B L  4BJ5C                130    0.055  10.107   0.165   0.831   0.044   0.125
B T  4BJ5C                131    0.147  20.417  -0.759   0.831   0.044   0.125
B C  4BJ5C                132    0.082  11.513  -0.649   0.751   0.050   0.199
B L  4BJ5C                133    0.069  12.616  -0.516   0.561   0.047   0.393
B S  4BJ5C                134    0.196  22.924  -0.771   0.268   0.043   0.689
B G  4BJ5C                135    0.182  14.292  -1.784   0.181   0.016   0.803
B D  4BJ5C                136    0.180  25.996  -0.998   0.184   0.043   0.773
B L  4BJ5C                137    0.059  10.803  -0.440   0.307   0.165   0.527
B M  4BJ5C                138    0.087  17.489  -0.444   0.321   0.252   0.427
B V  4BJ5C                139    0.047   7.285  -0.044   0.453   0.248   0.299
B F  4BJ5C                140    0.041   8.309   0.361   0.502   0.102   0.396
B P  4BJ5C                141    0.043   6.045  -0.481   0.717   0.014   0.269
B R  4BJ5C                142    0.197  45.136   0.023   0.879   0.010   0.111
B Y  4BJ5C                143    0.105  22.374  -0.239   0.923   0.002   0.076
B F  4BJ5C                144    0.030   5.961   0.269   0.923   0.002   0.076
B L  4BJ5C                145    0.030   5.493   0.172   0.938   0.007   0.055
B N  4BJ5C                146    0.140  20.525  -0.855   0.923   0.002   0.076
B M  4BJ5C                147    0.062  12.466  -0.055   0.858   0.002   0.139
B F  4BJ5C                148    0.058  11.641  -0.183   0.782   0.003   0.216
E K  4BJ5C                149    0.359  73.846   0.445   0.622   0.015   0.363
E D  4BJ5C                150    0.451  65.061  -1.149   0.339   0.016   0.645
E N  4BJ5C                151    0.330  48.297  -1.803   0.181   0.016   0.803
B V  4BJ5C                152    0.141  21.733  -1.592   0.058   0.017   0.925
E N  4BJ5C                153    0.389  56.876  -1.102   0.018   0.019   0.964
B P  4BJ5C                154    0.177  25.088   0.142   0.016   0.005   0.979
B P  4BJ5C                155    0.161  22.818  -1.172   0.016   0.005   0.979
E P  4BJ5C                156    0.333  47.224  -1.181   0.018   0.019   0.964
E N  4BJ5C                157    0.472  69.130  -1.518   0.018   0.019   0.964
B V  4BJ5C                158    0.068  10.375   0.068   0.018   0.019   0.964
B P  4BJ5C                159    0.184  26.166  -1.002   0.018   0.019   0.964
B G  4BJ5C                160    0.146  11.474  -2.149   0.019   0.141   0.840
B I  4BJ5C                161    0.101  18.759  -0.192   0.021   0.451   0.528
B W  4BJ5C                162    0.100  24.002  -0.035   0.021   0.451   0.528
B T  4BJ5C                163    0.244  33.857   0.280   0.022   0.359   0.619
E H  4BJ5C                164    0.428  77.817   0.159   0.064   0.216   0.721
E D  4BJ5C                165    0.616  88.737  -0.433   0.184   0.043   0.773
E D  4BJ5C                166    0.377  54.254  -0.599   0.339   0.016   0.645
E D  4BJ5C                167    0.366  52.784  -0.802   0.622   0.015   0.363
E E  4BJ5C                168    0.499  87.263   0.064   0.717   0.014   0.269
B S  4BJ5C                169    0.220  25.843  -0.070   0.717   0.014   0.269
B L  4BJ5C                170    0.112  20.580  -0.138   0.717   0.014   0.269
E K  4BJ5C                171    0.538 110.749   0.622   0.522   0.016   0.462
E S  4BJ5C                172    0.518  60.674  -0.406   0.115   0.016   0.868
E N  4BJ5C                173    0.568  83.199  -1.151   0.018   0.019   0.964
E D  4BJ5C                174    0.495  71.301   0.155   0.016   0.005   0.979
E Q  4BJ5C                175    0.684 122.091   0.169   0.858   0.002   0.139
E E  4BJ5C                176    0.713 124.631   0.447   0.858   0.002   0.139
B Q  4BJ5C                177    0.207  36.917  -0.372   0.858   0.002   0.139
B I  4BJ5C                178    0.043   7.881   0.108   0.923   0.002   0.076
E R  4BJ5C                179    0.458 104.928   1.885   0.938   0.007   0.055
E K  4BJ5C                180    0.375  77.137   1.597   0.938   0.007   0.055
B L  4BJ5C                181    0.048   8.844   0.436   0.938   0.007   0.055
B V  4BJ5C                182    0.163  25.068  -0.059   0.938   0.007   0.055
E K  4BJ5C                183    0.614 126.403   0.508   0.879   0.010   0.111
E K  4BJ5C                184    0.511 105.154  -0.041   0.717   0.014   0.269
E H  4BJ5C                185    0.350  63.610  -0.376   0.430   0.016   0.555
E G  4BJ5C                186    0.333  26.168  -1.797   0.113   0.043   0.844
B T  4BJ5C                187    0.234  32.497  -0.862   0.118   0.150   0.732
B G  4BJ5C                188    0.226  17.818  -1.611   0.216   0.235   0.548
E R  4BJ5C                189    0.280  64.212   0.694   0.373   0.401   0.226
B M  4BJ5C                190    0.145  29.055   0.061   0.487   0.370   0.144
E E  4BJ5C                191    0.357  62.368   0.258   0.594   0.285   0.121
B M  4BJ5C                192    0.106  21.151  -0.162   0.662   0.225   0.113
B R  4BJ5C                193    0.164  37.670   0.419   0.662   0.225   0.113
E K  4BJ5C                194    0.360  73.990   0.164   0.725   0.163   0.112
E R  4BJ5C                195    0.368  84.364   0.903   0.779   0.100   0.120
B F  4BJ5C                196    0.104  20.893   0.055   0.751   0.050   0.199
B F  4BJ5C                197    0.276  55.453   0.350   0.717   0.014   0.269
E E  4BJ5C                198    0.568  99.299   0.132   0.717   0.014   0.269
E K  4BJ5C                199    0.530 109.103   0.321   0.430   0.016   0.555
E D  4BJ5C                200    0.593  85.408  -1.360   0.181   0.016   0.803
E L  4BJ5C                201    0.282  51.707  -0.276   0.115   0.016   0.868
E L  4BJ5C                202    0.685 125.369  -1.286   0.003   0.003   0.994

Explain the output.  Go back.
```

---
